# The role of traditional Japanese medicine (Kampo) in the practice of psychosomatic medicine: the usefulness of Kampo in the treatment of the stress-related symptoms of women, especially those with peri-menopausal disorder

**DOI:** 10.1186/1751-0759-7-16

**Published:** 2013-10-22

**Authors:** Takahisa Ushiroyama

**Affiliations:** 1 Endowment Department of Mibyou Science and Medicine of Salutogenesis, Health Science Clinic, Osaka Medical College, Osaka, Japan

**Keywords:** Kampo medicine, Stress-related symptoms, Undefined symptoms, Pre-menopausal disorder, Tailor-made medicine

## Abstract

A serious problem currently plaguing the medical field is the widening gap between academic medicine, which studies the features and causes of illness, and the medical care that patients desire. An example of this gap can be observed in the practice of psychotherapy, which is effective only for certain patients. Kampo medicine that combines the advantages of Western medicine with those of traditional Japanese medicine is currently undergoing a revival in the healthcare sector. The therapeutic policies underlying Kampo medicine are based on the physical constitution and current symptoms of each patient. For this reason, Kampo medicine is referred to as “tailor-made medicine” and has properties similar to “mind and body” or psychosomatic medicine. Some women exhibit multiple undefined stress-related symptoms during the peri-menopausal period. In order to accurately diagnose and provide patient-specific treatment, physicians should not only investigate the various stress factors in patients’ lives but should also provide a Sho, or a Kampo diagnosis. The therapeutic approach in Kampo medicine is aimed at harmonizing the mind, body, and spirit; this practice involves the use of narrative and holistic medication that treats the entire being of the patient, resulting in an increased number of specialized treatment plans.

There are many Kampo prescriptions tailored to treat women who exhibit various stress-related symptoms. Both Kampo and psychosomatic medicine are based on the principles of narrative-based medicine, and by integrating these two medical systems, an ideal system can be devised to better cope with the various needs of patients. This new medical system established by integrating and harmonizing Western and Eastern medicine can be used for the treatment of women with stress-related symptoms.

## Introduction

Kampo medicine differs from Western medicine in terms of etiological concepts, methods of examination and evaluation of patients, diagnosis methods, and modes of selection of drugs for treatment of individual patients. These two systems of medical care have their own unique characteristics and complement each other.

In Western medicine, physicians use cellular pathology theories to simplify the diagnosis of illnesses and to ascertain a given pathologic condition. In contrast, in Kampo medicine, the practitioners make an effort to understand the complexity of the pathologic condition of a given patient (chaos) without simplifying the disease process [1].

However, both Kampo and psychosomatic medicine systems share a focus on holistic, tailor-made medicine. In particular, the fundamental concept of there being no distinction between the mind and body is characteristic to Kampo medicine and can easily be compared to the concepts of psychosomatic medicine, which state that there is a strong interrelation between mental and physical functioning in terms of life functions. In addition, Kampo theory proposes five *parenchymatous visceras* that govern all human psychological and physical experiences, which again closely mirrors the interrelationship between mental and physical functioning in psychosomatic medicine.

## Necessity and position of kampo in modern medicine

Technological and biomedical developments have modernized many aspects of our daily lives over the past century. However, these modern advances have also exposed people to significant stress, making mental and physical dysfunction more prevalent than in the past. Doctors have divided modern medicine into various small fields, and this division is primarily based on the organ targeted by the medicine. They normally investigate the morphological and quantitative abnormalities observed in the human body according to predefined criteria and base their findings on the mean values thus obtained. As a result, modern medicine is not effective in diagnosing or treating abnormal body functions and sometimes may not even diagnose the abnormal function as an illness [2].

Western clinical research involves calculating the mean values of the study subjects and standardizing the results while eliminating personal or individual biases as far as possible. However, according to one particular study, sick patients require medical care tailored to their characteristics, personality, and specific requirements [3].

Research groups who initially advocated evidence-based medicine have recently begun to shift their focus to narrative-based medicine and tailored medical care. Kampo medicine considers narrative-based medicine important (Table 1).

**Table 1:** Need for Kampo medicine in modern medical treatment

	
1.	There is now a major shift from HRT to various alternative medicine systems, such as Kampo, in the treatment of postmenopausal symptoms in the United States and elsewhere.
2.	Many aspects of daily life have undergone modernization over the past century, and today, individuals are exposed to significant mental and physical stress. Therefore, there is an increased risk of developing mental and physical dysfunctions.
3.	Modern medicine has been divided into various small fields based on the organ targeted by the medicine and usually assesses the morphological and quantitative abnormalities observed in the human body according to the criteria based on mean values.
4.	There is a large gap between academic medicine, which is based on the features and fundamental characteristics of diseases, and the medical care that patients actually wish to receive.
5.	When patients present diseases or symptoms that cannot be explained by modern Western medicine, doctors advise them that the illness is all in their mind, and they have the ability to endure it. Therefore, these patients remain untreated and concerned about the illness.

In modern society, physicians are required to determine if the medical care services rendered provide ease, comfort, and hope to sick patients and thereby improve their quality of life. In this regard, towards the end of the 19th century, Japanese traditional medicine was found to be more effective than Western medicine. However, the legislative and administrative systems underwent significant modifications during the Meiji Restoration. The Imperial Diet of 1895 stated that medical licenses would be given only to those individuals who passed the national examination on Western medicine. In other words, Kampo medicine was excluded from the public framework of medical systems in Japan. As a result, the number of physicians who prescribed traditional Kampo medicine rapidly decreased and treatment with Kampo medicine rapidly waned. However, a century later, in 2001, the Model Core Curriculum for Medical Education (Guidelines for Educational Contents) in Japan clearly stipulated that all medical students should know Japanese Kampo preparations, i.e., they should learn the essential requirements and have minimum knowledge about Kampo medicine. Thus, the traditional Japanese medical system was finally revived. Since then, an increasing number of universities have adopted the study of Kampo medicine as part of their pre-graduate medical curriculum. In 2004, 80 domestic medical universities offered courses on Kampo medicine.

Currently, the Japan Society of Oriental Medicine, which is responsible for the development of clinical skills and academic activities in Kampo medicine, comprises 8,570 members and 2,161 Kampo specialists across the country. The members of this society are doctors licensed to practice Western medicine, and they provide high-quality medical care by actively combining Kampo and Western medicines, thereby imparting the benefits of both systems to patients. The membership of the Japan Society for Oriental Medicine has been steadily increasing, with more than 3,000 members attending its annual meeting every year. This suggests that the foundation of Kampo medicine in Japan is getting stronger.

In the present age, various stress factors are responsible for many diseases. Therefore, the treatments for these diseases should involve both physical and mental interventions. In Kampo medicine, the mind cannot be treated separately from the body, because one of the fundamental principles of Kampo medicine is simultaneous treatment of the mind and body. Due to these characteristics, the demand for Kampo medicine has been gradually increasing [2]. The rapidly increasing demand for holistic medicine from patients with stress-related diseases may be one of the reasons for the official incorporation of Kampo medicine into the curriculum of medical education and the increase in the number of the members of the Japan society for Oriental Medicine.

## Suitability of kampo medicine in the treatment of peri-menopausal women with stress-related symptoms

Although women of all ages exhibit multiple undefined symptoms, they are particularly prominent in peri-menopausal women. While undefined symptoms during the climacteric period were commonly encountered in daily clinical practice, in Western medicine, interventions for these cases were limited to symptomatic therapy. Since estrogen deficiency is one of the reasons for the onset of various psychosomatic symptoms during the peri-menopausal period, estrogen replacement therapy has been commonly and widely practiced. However, there is no observable difference between stress-related symptoms and the symptoms caused by estrogen deficiency. Therefore, estrogen replacement therapy is not necessarily effective in all women experiencing these symptoms. To address these issues, clinical examinations should study the minds and bodies of these women, in addition to assessing the symptoms they experience. In other words, holistic medicine is necessary for peri-menopausal women exhibiting various undefined symptoms. There is historical evidence that supports the fact that Kampo medicine has treated multiple undefined stress-related symptoms. An ancient Kampo medicine textbook details various undefined symptoms and the respective Kampo formulations (Table 2).

**Table 2:** List of undefined symptoms and the respective Kampo formulation mentioned in ancient Kampo medicine textbooks

**Terms**	**Kampo formulation**
Restlessness arising from fear	Keishikaryukotsuboreitou Saikokaryukotsuboreitou
Restless feeling in the chest	Saikokeishikankyoto
Heavy sensation in the body like as mountain	Hangebyakujutsutennmatou
Fever accompanied by restlessness	Kamishoyosan
Fever and restlessness in the whole body	Seishinrenshiin Kamishoyosan
Asthenia of viscera, restlessness along with chronic consumptive disease	Sansoninto
A symptom-complex resulting from blood stagnation	Nyoshinsan
Feeling of a small food item obstructing the throat	Hangekoubokuto

In Kampo medicine, the concepts and disease states are explained by the conforming theories of holistic medicine and Yin-Yan, heat-cold, repletion-vacuity patterns or image of qi, blood, and fluids. This means that the diagnostic process and logic are based on the state of qi, blood, and fluids. The living body is considered to be a small universe and is treated based on phenomenology. Abnormal fluid metabolism (41.6%) and blood stagnation (39.5%) were detected as the main ailments in peri-menopausal women with headache, hot flashes, and dizziness as the main menopausal symptoms. Blood stagnation (36.5%), qi regurgitation (25.9%), and stagnation (24.8%) were detected in peri-menopausal women with undefined complaints during the climacteric period [4].

Certain problems encountered often in Western medicine cannot be resolved only with drug therapy, such as estrogen replacement. Various sociocultural problems cause stress-related symptoms in peri-menopausal women; such problems often disturb autonomic nerve function resulting in unpleasant symptoms (Table 3). Each menopausal woman with unpleasant symptoms exhibits non-specific responses to a pathological process because of her unique endocrinological condition, home and community environment, and personality even though she has no organic disorder. This concept is consistent with the theories underlying Kampo medicine. Like psychosomatic medicine, Kampo medicine is also a non-specific medical system.

**Table 3:** Stress factors in peri-menopausal women with undefined symptoms

**Investigation period**	**1994–1996**	**1997–2000**	**Significance**	**Odds ratio**
	**n = 284**	**n = 191**		
**Ratio of the consciousness of stress**	**70.0%**	**72.8%**		
	**199/284**	**139/191**		
Stress factors				
(Item)				
Conditional problems	55.8%	54.0%	NS	0.97
Workplace problems	11.1%	25.9%	P = 0.003	2.33
Spousal problems	23.6%	31.7%	NS	1.49
(Spousal relationship problems)	12.6%	22.3%	P = 1.146	1.77
Problems associated with children	19.6%	35.3%	P = 0.014	1.80
(Attitude problems and concerns about the prospective life of the child [children])	10.1%	22.3%	P = 0.008	2.21
Parental problems	16.6%	24.5%	NS	1.48
Problems associated with the nursing of parents	8.0%	15.1%	P = 0.048	1.89

Some clinical reports have revealed that Kampo preparations improve human clinical conditions associated with menopause [5-9]. Samukawa demonstrated that Dan-gui-shao-yao-san combined with red ginseng powder improved climacteric disorder symptoms [10]. Like dan-gui-shao-yao-san, gui-zhi-fu-ling-wan (keishibukuryogan), a preparation often used to treat peri-menopausal disorders, is known to be more effective when used in combination with tofisopam, a Western drug [11].

A comparative study between Kampo and hormone replacement therapy (HRT) revealed differences in therapeutic effects. It was reported that HRT was more effective for hot flashes, perspiration, depression, and insomnia, whereas Kampo therapy was more effective for malaise and chills [12]. In our previous study, Keishibukuryogan significantly increased the blood flow in the toes, resulting in a decrease in hot flashes in the upper part of the body and relief of cold sensation in the lower extremities of postmenopausal women, whereas HRT decreased the blood flow to the lower extremities [13].

Similar results in comparative studies between Kampo therapy and HRT, as well as in studies on the effects of the combined treatment with Kampo and HRT, have been demonstrated in peri-menopausal women with depression and anxiety. Higuchi et al. [14] demonstrated that the Hamilton anxiety score was significantly lower in peri-menopausal women with undefined symptoms undergoing treatment with Kamishoyosan (Jia-ewi-xiao-yao-san) than in those treated with HRT. No significant differences were observed in the self-rating depression scale (SDS) scores, the Pittsburg sleep-quality index, and the climacteric symptoms rating scale (developed by the Japan Society of Obstetrics and Gynecology) scores between the treatment groups. Matsuo [15] revealed that the combined treatment with Unkeito (Wen-jing-tang) and HRT significantly decreased the SDS score and significantly improved the state and trait anxiety index (STAI).

The primary objective of Kampo medicine, as a biological response modifier (BRM), is to restore the patient’s physiological environment by regulating the physiological balance of the neurological, endocrinological, and immunological systems, rather than directly targeting affected cells or organs [16,17]. Kampo medicines function as neurotransmitters, immunomodulators, or endocrine modulators [16]. Chen demonstrated that Keishi-bukuryo-gan decreased hot flashes associated with plasma calcitonin gene-related peptide (CGRP) level reduction [7]. Koike revealed that administration of Unkeito for 3 months significantly improved ZSDS and STAI-1, 2 levels [18]. Our recent studies demonstrated that kamishoyosan reduced plasma interleukin (IL)-6 and soluble IL-6 receptor levels and increased plasma TNF-α level in depressive peri-menopausal women [19,20] (Table 4).

**Table 4:** Changes in plasma IL-6, sIL-6R, and TNF-α concentrations in depressed menopausal patients with undefined symptoms after treatment for three months with Kami-shoyo-san or anti-depressants

	**Kami-shoyo-san**		**Anti-depressants**		**Significance**
IL-6					
% change	−29.1 ± 7.3%		8.6 ± 6.5%		P < 0.0001
plasma level					
before treatment	1.88 ± 1.56 pg/ml	]*1	2.06 ± 1.99 pg/ml	]*3	NS
After 3-month treatment	1.20 ± 0.85 pg/ml		2.35 ± 3.79 pg/ml		P = 0.04
					
sIL-6R					
% change	−17.6 ± 4.9%		3.4 ± 6.1%		P = 0.001
plasma level					
before treatment	104.3 ± 25.5 ng/ml	]*2	99.8 ± 29.5 ng/ml	]*4	NS
3-months treatment	92.0 ± 33.7 ng/ml		102.4 ± 32.7 ng/ml		NS
					
TNF-α					
% change	21.3 ± 5.4%		−6.8 ± 2.2%		P < 0.001
plasma level					
before treatment	14.2 ± 6.3 pg/ml	]*5	15.0 ± 6.6 pg/ml	]*6	NS
3-months treatment	17.2 ± 6.1 pg/ml		14.5 ± 6.0 pg/ml		P < 0.05

*1: P = 0.02, *2: P = 0.02, *3: NS, *4: NS, *5: P < 0.0001, *6: NS.

## Psychosomatic medicine for the treatment of peri-menopausal women with stress-related symptoms, especially those with empty-nest syndrome

The maturation of a mother-child relationship is a developmental process that needs to be worked through by mothers and their children if the children are to achieve satisfactory psychological development and the mothers are to cope with their changing maternal roles. Some women develop complaints during the peri-menopausal period due to unidentifiable causes. In 9.0% of women experiencing menopause, the complaints can be attributed to the “empty-nest syndrome,” i.e., psychologically, they have been unable to come to terms with the fact that their active role as a mother is over [21]. In 26.7% of peri-menopausal women, the psychological stress caused by unsatisfactory relationships within the family is found to be the cause of the complaints. In 32.1% of these cases, the patients’ relationship with their children is the source of stress. The stress related to failed relationships within the family or society is amplified by a depressive character style (typus melancholicus), introversion, self-sacrifice, domestic martyrdom, or alexithymia, leading to various mental and physical symptoms that some researchers have grouped together as the unidentified complaint syndrome [22].

In recent years, Western mental health professionals have increasingly focused on and collected evidence of Asian psychologies, suggesting that the potential contributions of these psychologies may have been underestimated. Michalon reported that Buddhist psychology has now gained some credence in the West and is beginning to exert a growing influence both on various areas of medicine as well as on well-established Western psychotherapies [23].

Peri-menopausal patients with unidentified complaint syndrome (including mental disorders) are often exposed to various mental stress factors associated with complex social environments. These patients are often thrust into environments where no support is extended from people around them, and they often remain silent without expressing their agony to those around them. Nowadays, factors that often cause mental stress to middle-aged women are their health, relationship with their partners, and parental care [4]. In recent years, the percentage of middle-aged women exposed to mental stress due to their children has been increasing. The percentage ascertained by the 1997–2000 survey was 4.7 times higher than that obtained in the 1994–1996 survey. During the same period, there was a 2.9-fold increase in the percentage of women exposed to mental stress due to spousal relationships. These changes probably indicate that the aging trend and decrease in the number of children in the Japanese society in recent years is reflected in the psychosomatic disease profiles of middle-aged women.

In Japan, Morita therapy has been used for the treatment of neurosis or certain psychosomatic diseases, including climacteric disorder. Unlike cognitive-behavior therapy, Morita therapy is not aimed at eliminating the symptoms. Peri-menopausal women are likely to develop unidentified complaint syndrome triggered by exposure to stress associated with spousal relationships, parents (due to their daily living care), and relationship with their children. Often, children take priority, and for many women, their primary concern is their children during their daily life. It should be noted that souring of relationships with their children or having an extremely close relationship with their children can cause physical and mental disturbances in these women, which sometimes may require psychosomatic medical treatment. When dealing with these women, diverse clinical measures may be required, including temporarily diverting the woman’s concern from her child and administering five parenchymatous viscera as mentioned in Kampo medicine theory-based psychotherapy.

## Sho as a stress reaction and practical kampo therapy

In Kampo medicine, the findings of abdominal examination are interpreted in terms of the distribution of tension, resistance, and tenderness of the abdominal wall, which are the underlying pathological causes that manifest as clinical signs. Traditionally, the therapist used to identify these findings using his five senses; however, recently, there have also been attempts to objectify these findings.

The outcome of Kampo treatment of abdominal conditions presents a holistic picture of patients’ symptoms and psychosomatic conditions of women around the globe. Two herbal medicines, *kamishoyosan* and *saikokeishikankyoto*, are commonly used to treat stress-related symptoms in women, especially in peri-menopausal women. Kampo herbal medicine preparations are administered according to the patient’s physical constitution and disease progression taking into account his/her resistance to the disease. Therefore, after Kampo diagnosis of an abdominal condition, therapy involves abdominal palpation. The abdominal pattern called “Fukusho” integrates many of these elements along with the consistency and resistance of the abdominal wall. Therefore, “Fukusho” as one of the clinical findings of traditional Japanese Kampo indicates objective figures that are related to pathologic signs.

**Figure 1:**
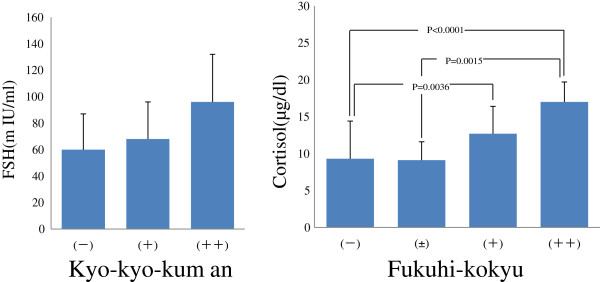
Correlation of plasma FSH and cortisol levels with abdominal palpation findings.

Recently, we tried to identify normal abdominal patterns observed in patients with multiple stress-related symptoms using an AXIOM vibration sensation sensor to measure the consistency and resistance of the abdominal wall at a subcutaneous depth of 8–10 mm. This abdominal examination was performed in 289 middle-aged or elderly women with unidentifiable symptoms who visited our clinic. During the examination, blood levels of follicle stimulating hormone (FSH), luteinizing hormone, and estradiol were determined using enzyme immunoassays. The vibrator sensor values for consistency and resistance of the abdominal wall were found to be significantly higher in patients presenting with a marked degree of Kyokyo Kuman (fullness, tenderness, or discomfort of the hypochondrium), Fukuhi Kokyu (tense abdominal skin and rectus abdominis muscle contraction), and Shofuku Kyuketsu (distention and cramping of the lower abdomen), p < 0.01, p < 0.05, and p < 0.05, respectively. In addition, with increased Saika Fujin (subumbilical insensitivity) severity, the values for consistency and resistance of the abdominal wall tended to be significantly less (1+: p < 0.05; 2+: p < 0.0 l). In patients with Kyokyo Kuman complaints, the blood FSH levels were found to be significantly higher (66.3 ± 25.7 mIU/ml) than in those devoid of this symptom (59.8 ± 18.4 mIU/ml). A positive correlation between Kyokyo Kuman severity and plasma FSH concentration indicates qi stagnation, which is a manifestation of an anomaly in qi, blood, and water metabolism regulation according to Kampo medical pathology. This could also indicate a possible correlation with the aging of the reproductive system [24]. This condition often develops due to continuous mental distress or unresolved problems. In other words, Fukusho has a possible correlation with stress. A strong correlation was also observed between the degree of Shofuku Kyuketsu and cortisol concentration in the blood; cortisol blood concentration levels are significantly higher in symptomatic patients (13.5 ± 3.6 μg/dl) than in asymptomatic patients (8.8 ± 4.7 μg/dl) (p < 0.000 l and p = 0.0376, respectively) (Figure 1). Cortisol, an indicator of physical stress, also has a strong correlation with Fukuhi Kokyu (tense abdominal skin and rectus abdominis muscle contracture). These results suggest that abdominal palpation during Kampo medical examination may allow the detection of asymptomatic stress-related symptoms. This could also be stipulated as a criterion for prescribing preparations containing crude extracts, such as “Saiko” or “Ninjin”, which are considered to possess anti-stress properties, for the treatment of Fukuhi Kokyu. Moreover, administration of appropriate Kampo medicines can possibly prevent stress-induced diseases that might develop in the future. Normally, certain Kampo preparations for the treatment of qi stagnation and counterflow are considered suitable for the treatment of stress-related psychosomatic disorders.

We tried to objectify the abdominal examination findings that are directly related to the selection of the appropriate prescription and demonstrated the possibility of expressing these findings in a numerical format. The results suggest that the findings could reflect the magnitude of endocrine aberrations. Moreover, these results also indicate that the abdominal examination findings in one of the Kampo medical examinations could possibly convey information about the condition of the endocrine environment and the state of the autonomous nervous system. These are considered to be important indices for selection of the appropriate therapy, and these indices vary among individuals. We believe that a larger sample size and further detailed investigations are required.

## Direction of medical services in the integration of kampo and psychosomatic medicine

Kampo medical textbooks contain over 4,000 years of clinical data and literature. These textbooks detail valuable diagnostic criteria for practicing medicine tailored to meet the requirements of individual patients. Therefore, Kampo medicine not only uses the five senses to diagnose patients (such as women suffering from stress-related symptoms) but is also a medical system that aids the practitioner in acquiring a healing mind, which is one of the basic aspects of this medicine system. Therefore, by integrating Kampo with psychosomatic medicine, which is a branch of modern Western medicine, we may be able to create an ideal medical system to meet the various medical requirements of patients. A new system of medicine can be established by integrating and harmonizing Western and Eastern medical practices for the treatment of women with stress-related symptoms.

Kampo medicine emphasizes the importance of the use of the five senses in diagnosis; however, a further understanding of the pathological states mentioned in the Kampo theory is warranted [3,25-27].

Herbs are believed to affect both the psyche and soma, and Kampo medicine does not differentiate between the two. Currently, the incidence of stress-related diseases is increasing. The relationship between the seven emotions, as an endogenous pathogenic factor, and stress-related physical symptoms in Kampo medicine correspond to the mind-body correlation in psychosomatic medicine. Furthermore, in Kampo medicine, extreme changes in emotions and pleasure affect physical functions; they can also manifest as pathological stress symptoms. Sometimes, stress-related symptoms in women manifest as wounds, disorders, or imbalances in the five *parenchymatous viscera*. Treatment of such diseases requires both mental and physical interventions. In particular, without considering the involvement of mental factors, it may be difficult to treat diseases in women, and therefore, holistic approaches are desirable. In Kampo medicine, the mind cannot be considered separate from the body during the treatment process; simultaneous treatment of the mind and body is one of the fundamental principles of Kampo medicine. Due to these specific characteristics of Kampo medicine, there is an increase in the demand for Kampo medicine. Furthermore, this fundamental principle is consistent with that of psychosomatic and integrated medicine; this suggests that Kampo medicine can play a significant role in the practice of psychosomatic medicine. Modernization and civilization of human life has led to increased exposure to mental and physical stress, which in turn has increased the likelihood of people developing mental and physical dysfunctions. Therefore, the increased likelihood of psychosomatic disease, particularly functional disorders (e.g., undefined complaints) is a characteristic of this era.

Tonic formulations responsible for Hochuekkitou and Juzendaihotou contain Radix Ginseng and are often prescribed for women with multiple stress-related symptoms. Ancient Chinese medical textbooks state that Radix Ginseng can reduce anxiety and increase resistance to stress. Such formulations are essential for a society in which people are exposed to various forms of stress. Furthermore, certain herbs, such as Paeoniae Radix and Magnoriae Cortex, have calming effects; formulations with these herbs, such as Tokishakuyakusan, Kamishoyosan, Keishikaryukotsuboreitou, and Hangekobokuto, are frequently prescribed to women who have been diagnosed with psychosomatic disease. Formulations containing Saiko, which is an herb that alleviates stress, such as Kamishoyosan, Saikokeishikankyoto, Yokukansan, and Saikokaryukotsuboreito, are frequently prescribed to women with stress-related symptoms.

With regard to the treatment for these diseases, the need of the hour is tailor-made medical care that focuses on the characteristics and personality of each individual. Such medical care may also include psychotherapy, which is often effective only in certain patients. Tailored treatment approaches and narrative-based medicine are the main features of both Kampo and psychosomatic medicine.

## Competing interests

The author has competing interests in some companies as follows in this article: FamilyMart Co., Ltd., ROHTO Pharmaceutical Co., Ltd., PARAMOUNT BED Co., Ltd., FUJITSU LIMITED, FUJIFILM Medical Co., Ltd., The Zenitaka Corporation, KSK CO., LTD., NIPPON TELEGRAPH AND TELEPHONE WEST CORPORATION, MEDICEO CORPORATION, Asami CO., LTD., Godotoho Co., Ltd., KURAYA SANSEIDO Inc., PARATECHNO CO., LTD., Konishi Medical Instruments Co., Ltd., Tsumura & Co..
